# “Sandwich” protocol based on modified SMILE regimen for children with newly extranodal NK/T cell lymphoma, nasal type: a single-arm, single-center clinical study

**DOI:** 10.1007/s00277-023-05375-3

**Published:** 2023-07-24

**Authors:** Cheng-qi Shen, Guo-qian He, Zhi Wan, Chao Lin, Xue Yang, Xiao-xi Lu, Yi-ping Zhu, Ju Gao, Xia Guo

**Affiliations:** 1 Department of Pediatrics, West China Second University Hospital, Sichuan University, Section 3, South Renmin Road, Chengdu, 610041 China; 2 NHC Key Laboratory of Chronobiology, Sichuan University, Chengdu, 610041 China

**Keywords:** Non-Hodgkin’s lymphoma, Extranodal NK/T-cell lymphoma, Sandwich therapy, Children

## Abstract

Extranodal NK/T-cell lymphoma, nasal type (ENKTL), which is a rare form of mature T/NK cell lymphoma in children, currently lacks a standardized first-line treatment approach. However, a treatment protocol known as the “sandwich” regimen has been used in children newly diagnosed with ENKTL. This protocol combines the administration of methotrexate, ifosfamide, etoposide, pegaspargase, and dexamethasone (referred to as SMILE) with the addition of radiotherapy (RT). From September 2017 to December 2020, a total of five patients were included in the study, consisting of three males and two females. The median age of onset was 10.6 years (range, 9.8 to 14.0 years). Among the patients, four had nasal/nasopharyngeal disease at stage II, while one patient had extra nasal disease involving the skin at stage IV. The median EBV-DNA level in plasma was 1.68 × 10^3^ copies/ml (range, 0.44 to 21.1 × 10^3^copies/ml). All the patients had good overall response after 2 cycles of chemotherapy and radiotherapy, including 4 of the patients who had a complete response and 1 of the patients with partial remission. The patient with stage IV received allogeneic hematopoietic stem cell transplantation after the EBV-DNA level was elevated again during treatment. One patient in the low-risk group experienced grade 4 oral mucositis, while no other severe complications or treatment-related deaths were observed. The median follow-up period was 22 months (range, 5 to 57 months). All five patients successfully completed their treatment, with four patients achieving event-free survival, and one patient was lost to follow-up. The median OS time and EFS time was 33 months (range: 18–57 months) and 20 months (range: 5–47 months), respectively. The sandwich protocol has demonstrated a high response rate, good tolerance to chemotherapy, and no treatment-related fatalities. However, further confirmation is necessary through additional clinical studies involving larger sample sizes. Clinical trial registration number: Due to modified SMILE regimens with sandwiched radiotherapy yielded promising outcomes in children ENKTL, we have carried out a phase II multicenter clinical trial (ChiCTR220005954) for children ENKTL in China to further verify the efficacy and safety.

## Introduction

Extranodal NK/T-cell lymphoma (ENKTL, previously ENKTL, nasal type) is a rare subtype of mature T/NK cell neoplasms in the 5th edition of the World Health Organization classification of hematolymphoid tumors in 2022 (WHO-HAEM5) [1–4]. ENKTL is characterized by universal extranodal involvement and is closely associated with invariable Epstein-Barr virus (EBV) infection [5, 6]. According to the SEER database of the National Cancer Institute, the incidence of ENKTL is about 0.7 cases per 1,000,000 people [7]. ENKTL is more prevalent in Asia (such as China, Japan, and South Korea) and central and South America (such as Mexico, Peru, Brazil), accounting for 5–15% of NHL in the above regions [8]. ENKTL primarily affects adult males, typically presenting with a median onset age of 50 years. However, there is a lack of precise clinical epidemiological data regarding its occurrence in children [9–11].

ENTKL exhibits an aggressive nature and typically manifests with locoregional invasion, resulting in necrosis, the formation of masses, obstruction, or hemorrhage. More than 80% of ENKTL cases involve the nasal cavity, including the nasopharynx, oropharynx, oral cavity, hypopharynx, various parts of the aerodigestive tract, and Waldeyer’s ring. Although rare, there are also instances of ENKTL occurring in extranasal sites such as the skin, soft tissue, testis, gastrointestinal tract, and reproductive organs [9]. These cases may also involve a small number of newly diagnosed cases and a large number of relapsed or refractory cases, which have a worse outcome. The underlying mechanisms and molecular drivers of ENKTL’s pathobiology remain unidentified. Newly available data has shown the presence of recurring mutations, copy number variations, and epigenetic alterations, specifically hypermethylation, in various genes associated with ENKTL [12]. However, data about the genetic alterations of the disease are limited due to its rarity and the commonly observed extensive tumor necrosis [13].

ENTKL poses significant challenges in terms of treatment and has historically been associated with an unfavorable prognosis. The standard treatment approaches have typically involved a combination of surgery, chemotherapy, and radiotherapy (RT). The 5-year overall survival (OS) rate for patients treated with these modalities has been approximately 50%. ENKTL patients with relapsed or refractory disease have dismal outcomes, with median survival of less than 12 months [14, 15]. Currently, there is a lack of established standard first-line treatment for ENKTL, particularly in children, despite the conduct of numerous prospective studies exploring novel drugs and showing improved patient outcomes [16, 17]. Hence, there is a critical requirement to establish an effective frontline treatment approach for ENKTL [4]. RT combined with chemotherapy is the most widely used treatment for ENKTL [8]. Moreover, there is a scarcity of data concerning the treatment and outcomes of younger patients, and treatment protocols designed for adults are frequently employed as a reference in these cases. The SMILE therapy is associated with overall response rates (ORRs) ranging from 60 to 80% [18]. However, SMILE has resulted in a high level of therapeutic compliance in adult. In comparison to adults, children generally have fewer underlying diseases and exhibit greater tolerance to chemotherapy. Consequently, this study aimed to investigate the effectiveness and safety of the sandwich protocol, which is based on a modified SMILE regimen, specifically designed for children with ENKTL.

## Materials and methods

### Trail patients

Clinical outcomes were analyzed for eligible patients treated at West China Second Hospital, Sichuan University, from September 2017 to December 2020. Patient medical records were retrospectively reviewed and collected if they identified with suitable disease names (e.g., “lymphoma”). The resulting datasets contained five ENKTL cases. Patients (≤ 18 years of age, female or male) with histologically confirmed NKTCL who had no treatment and expected survival time > 3 months were recruited into this trial. The diagnosis of ENKTL, nasal type was histologically confirmed in all of patients according to the criteria of the current WHO Classification of Tumours of Hematopoietic and Lymphoid Tissues (2017) [8, 19]. Tissue biopsy should be performed in all patients before chemotherapy. For patients with both extranasal and intranasal lesions, nasal mass biopsy should be taken at the same time. The molecules investigated in the tumor tissue for differential diagnosis include NK cell-related antigens (CD2, CD56, cytoplasmic CD3), T-cell antigens (CD3, CD4, CD5 and CD8), B-cell antigens (CD19, CD20, and CD79a), and cytotoxin molecules: perforin, granzyme B and T cell-restricted intracellular antigen-1 (TIA1) [20]. In situ hybridization for EBV-encoded RNA (EBER) was performed and should be positive for all patients. Key exclusion criteria encompassed NK cell leukemia, precursor NK cell neoplasms, any clinical conditions involving organ dysfunction unrelated to the disease (such as severe mental, nervous, cardiovascular, and respiratory system disorders), and patients who had previously undergone alternative chemotherapy regimens.

All patients gave their written informed consent before their enrollment to the study. The protocol was approved by the ethics committees at West China Second Hospital of Sichuan University on September 2017 and followed the Declaration of Helsinki principles [21].

### Trial design, treatment, and procedures

This trial was conducted as a single-arm, open-label, non-randomized study, meaning that male and female patients were not assigned to different groups through randomization. Computed tomography (CT), magnetic resonance imaging (MRI), and positron emission tomography (PET)/CT were performed based on the tumor-involved site before chemotherapy. Lumbar puncture, bone marrow smear, and biopsy were performed to determine whether tumor involved central nervous system and bone marrow in all the patients. Plasma EBV-DNA copy number was monitored by real-time quantitative polymerase chain reaction (RT-PCR) before treatment. Ann Arbor stage system was used for staging [22]. Patients were stratified by the revised prognostic index of ENKTL-PINKE [23] into 3 groups, G1, G2 and G3, with escalation of treatment intensity in ascending order (Table 1).

**Table 1:** The revised PINK-E scoring criteria

Factors	PINK-E
Stage III-IV	1
Distant lymph node involvement	1
ENKTL, non-nasal type	1
detected EBV-DNA in plasma	1

G1 (low risk): 0–1; G2 (medium risk): 2; G3 (high risk): ≥ 3

**Fig. 1:**
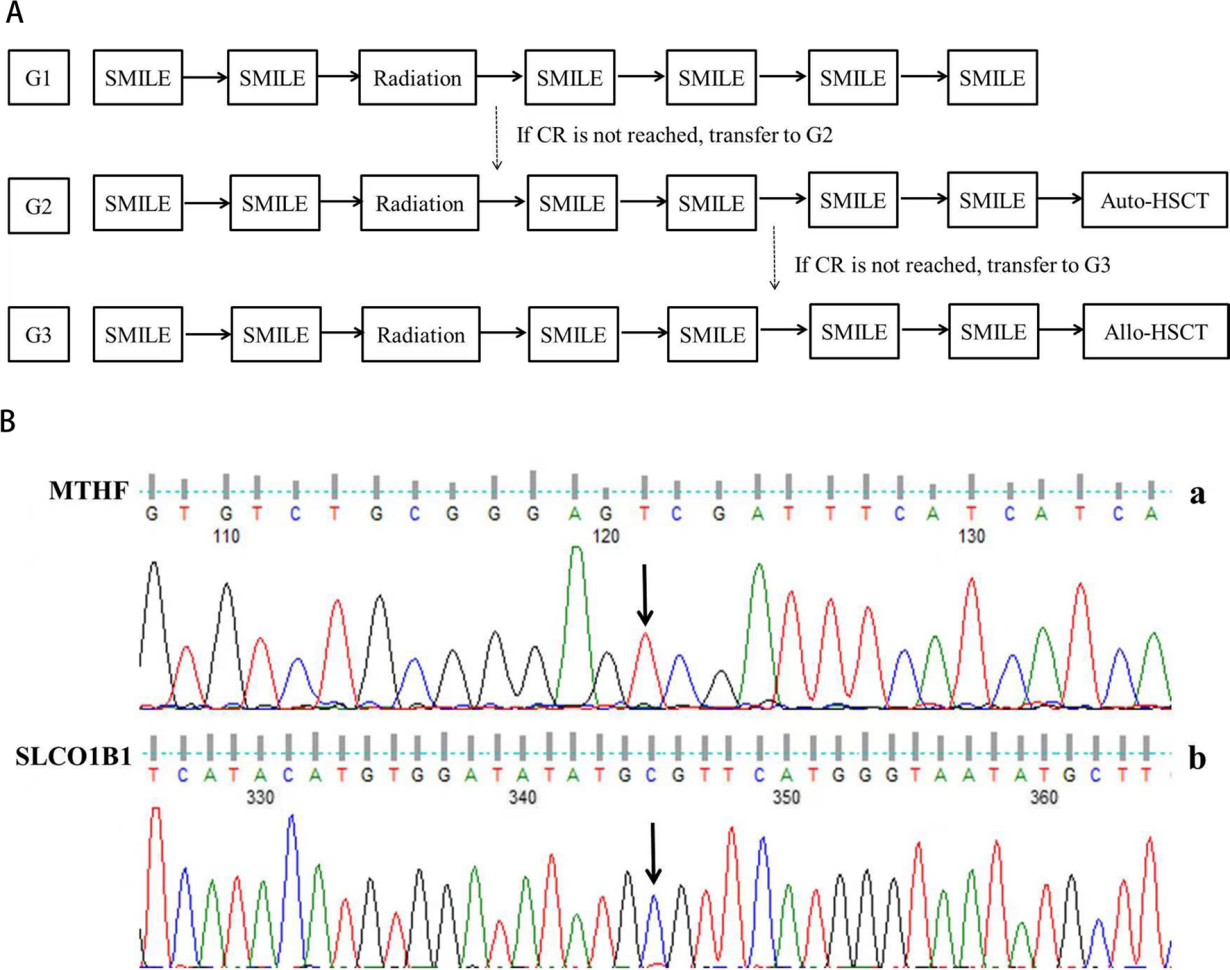
**A** Treatment flow chart of ENKTL. **B** The patient had homozygous mutation site in *MTHFR* (C677T) and *SLO1B1* (rs4149056) in case 3

The treatment scheme is shown in Fig. 1A. Patients underwent treatment on a 21-day cycle, receiving one of three chemotherapy backbones determined by the revised prognostic index. Following the second cycle of chemotherapy, RT was initiated, delivering a total dose of 50.4 Gy in 28 fractions. The RT was administered once a day, with five fractions administered each week. One week following completion of RT, chemotherapy was resumed for two to four cycles. The doses and administration schedule of the drugs used are detailed in Table 2. The dose of chemotherapy was decreased by one dose level for ANC < 0.5 × 10^9^/L lasting for > 7 days, ANC < 0.5 × 10^9^/L with sepsis, and any other drug-related non-hematological grade 3 or 4 toxicity or inability to tolerate one course of therapy due to toxicity.

**Table 2:** Drugs’ doses and administration schedule

Drug	Dose per day	Schedule
Methotrexate	2 g/m^2^, ivgtt (6h)	d1
Calcium tetrahydrofolate	15 mg/m^2^ × 4 doses, iv	d2-4
Ifosfamide	1500 mg/m^2^, ivgtt (> 1 h)	d2-4
Mesna	400 mg/m^2^, iv (0 h, + 4 h, + 8h)	d2-4
Dexamethasone	23 mg/m^2^, iv	d2-4
Etoposide	100 mg/m^2^, ivgtt (> 3 h)	d2-4
PEG-ASP	2000 μ/m^2^, im	d8
G-CSF		d6

Safety assessments were conducted on days 1, 8, 15, and 21 of the first cycle, and on days 1 and 8 of subsequent cycles. Definition and grading standards of adverse reactions were referred to the Common Terminology Criteria for Adverse Events version 5.0 developed by American National Cancer Institute [24]. Peripheral blood, including peripheral blood ANC and mononuclear cells, was collected on cycle 1 day 1 (pre-treatment) and cycle 1 day 8 (end-treatment). Patients were monitored for various factors that could impact prognosis and long-term side effects, such as age, gender, treatment response, serum lactate dehydrogenase levels, plasma EBV-DNA levels, presence of B symptoms, regional lymph node involvement, and Ann Arbor stage. Treatment response classified as complete response (CR), partial response (PR), or stable disease (SD) was assessed by non-Hodgkin’s lymphoma Efficacy Evaluation Criteria [25]. Disease assessment using cross-sectional imaging (CT or PET-CT) was conducted at the beginning of the study and then repeated every three cycles thereafter.

### Statistical methods

All patients were followed up to June 30, 2022. The primary end points were CR, PR, and ORR (ORR = CR + PR) after 2 treatment cycles. The secondary end points were overall survival (OS) time, event-free survival (EFS) time, and toxicity. OS was defined as the duration from enrollment to either the end of the follow-up period or the occurrence of death. EFS referred to the period from diagnosis to disease progression, relapse, the development of a secondary tumor, death from any cause, or the last recorded follow-up.

## Results

### Patients and basic characteristics

Five patients with newly diagnosed ENKTL were enrolled. As shown in Table 3, the median age was 10.0 years old (range: 9.8 to 14.0 y). There were 3 male patients and 2 female patients with sex ratio of 1.5. The median time to diagnosis was 4 months, with a range of 2 to 14 months. Among the cases, four were classified as stage II, while one case was classified as stage IV. Three of the cases exhibited B symptoms. Based on the revised prognostic index of ENKTL (PINKE), four patients were categorized into the G1 group, while one patient fell into the G2 group.

**Table 3:** Clinical features of individuals with ENKTL

ID	Case 1	Case 2	Case 3	Case 4	Case 5
Gender	Female	Male	Female	Male	Male
Disease-onset age	9.8	9.8	14.0	10.6	12.3
Type	Nasal type	Nasal type	Nasal type	Nasal type	Nasal type
Involved site	Skin, nose, bone, bilateral cervical and inguinal lymph nodes	Nose, anterior nasal cavity, palatine tonsil, adenoid	Left maxillofacial region, nasal root, inner canthus of left eye, left middle lower nasal meatus	Posterior part of nasal septum, left nasal cavity	Nasal septum, lateral nasal wall, hard palate
EBV-DNA (copies/ml)	Negative	1.91 × 10^3^	2.11 × 10^4^	666	440
LDH (U/L)	Normal	Normal	Normal	Normal	Normal
Stage	IV	IIE	IIE	IIE	IIE
Risk group	G2	G1	G1	G1	G1

#### Case 1

**Fig. 2:**
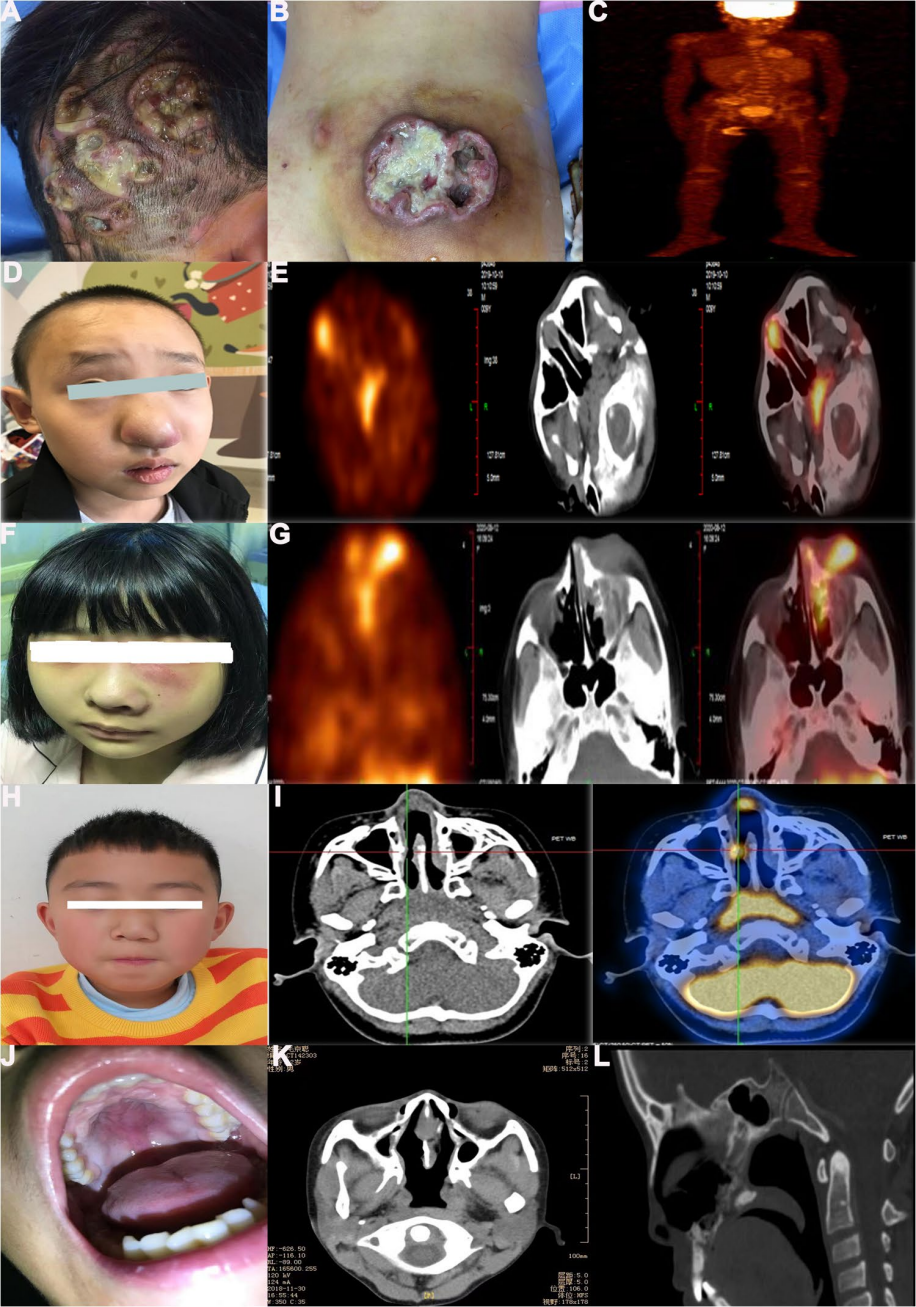
Case 1: Skin ulcers with yellow and white secretions covered on the head (**A**) and right lumbosacral region (**B**). PET/CT revealed that tumor involved in multiple skin and subcutaneous tissues, nose, bone, bilateral cervical and inguinal lymph nodes (**C**). Case 2: Alae nasi of the patient was red and swollen (**D**). The PET imaging demonstrated that tumor involved in nose, anterior nasal cavit, palatine tonsil, adenoid (**E**). Case 3: The patient had left maxillofacial redness and swelling, left alar nasi swelling (**F**). Serum EBV-DNA load was 1.91 × 10^3^copies/ml. The PET imaging showed that tumor involved in the left maxillofacial region, nasal root, inner canthus of the left eye and left middle and lower nasal meatus (**G**). Case 4: The patient had the left nasal alar and left nasal mass (**H**). The PET imaging showed the tumor involved in the posterior nasal septum and the right nasal cavity (**I**). Case 5: Mucosa of the midline upper jaw was red and swollen (**J**). Nasopharynx enhanced CT imaging showed that soft tissue thickening of nasal septum and lateral nasal wall, partial discontinuity of nasal septum bone **(K**), and irregular bone destruction in hard palate and upper alveolar (**L**)

A 9-year-and-9-month-old girl was referred to our department for repeated skin ulcers over a year and nasal mass over 2 months, accompanied by purulent nasal secretions and nasal peculiar smell. She had been diagnosed with “impetigo,” which could be improved after anti infection treatment. Due to aggravation of skin ulcers and new nasal mass, biopsy was performed in West China Hospital. A physical examination showed that multiple ulcers were scattered all over the body (Fig. 2A, B). Some ulcers exhibited a scabbed center with raised periphery, resembling a crater shape. Additionally, yellow and white secretions were visible on the surface of some ulcers. Plasma EBV-DNA was negative. PET/CT revealed that tumor involved in multiple skin and subcutaneous tissues, nose, bone, bilateral cervical, and inguinal lymph nodes (Fig. 2C). Based on these findings, the diagnosis of ENKTL with medium-risk stage IV disease was confirmed.

#### Case 2

A 9-year-and-9-month-old boy was admitted for nasal mass with pain over 3 months. As the pain gradually worsened, accompanied by redness and swelling of the right nasal alar, a biopsy of right nasal mass was performed, and the diagnosis of ENKTL was confirmed by pathology. During the physical examination, it was observed that the alae nasi were inflamed and swollen, accompanied by the presence of a nasal odor (Fig. 2D). Plasma EBV-DNA load was 1.91 × 10^3^ copies/ml. The PET imaging demonstrated that tumor involved in nose, anterior nasal cavity, palatine tonsil, and adenoid (Fig. 2E). The patient was diagnosed to have ENKTL with low-risk stage II.

#### Case 3

A 14-year-old girl suffered from swelling of the left face for more than 2 months, accompanied by recurrent high fever and nasal obstruction. Due to the progressive aggravation of facial swelling, a maxillofacial MRI was performed and found a mass in the left nasal cavity. She underwent a biopsy and was admitted to our department after the diagnosis being confirmed by pathology. The physical examination revealed redness and swelling on the left side of the maxillofacial region, as well as swelling of the left alar nasi (Fig. 2F). Plasma EBV-DNA load was 1.91 × 10^3^copies/ml. The PET/CT showed that the left maxillofacial region, nasal root, inner canthus of the left eye, and left middle and lower nasal meatus were invaded by tumors (Fig. 2G). Based on the aforementioned findings, a diagnosis of low-risk stage II ENKTL was established.

#### Case 4

The patient, a 10-year-and-7-month-old boy, experienced nasal obstruction and purulent discharge for a duration of 5 months. Two months prior to admission, a nasal mass with an odor was discovered in the patient. Subsequently, a biopsy of the nasal mass was conducted, confirming the diagnosis of ENKTL through pathological examination. A physical examination revealed enlargement of the left nasal alar and left nasal mass (Fig. 2H). Plasma EBV-DNA load was 666 copies/ml. The PET/CT scan revealed the tumor’s involvement in the posterior nasal septum and the right nasal cavity (Fig. 2I). Based on the above results, the diagnosis of ENKTL with low-risk stage II disease was drawn.

#### Case 5

The patient is 12-year- and-3-month-old ethnic Yi boy. He suffered from nasal odor and obstruction for 4 months, accompanied by purulent nasal discharge, recurrent high fever, and emaciation. Mass was found in nasal cavity by nasal endoscopy, and biopsy was performed. The patient was diagnosed with ENKTL based on pathological examination and subsequently referred to our department. During the physical examination, the nasal appearance appeared normal, but there was a significant presence of purulent discharge within the nasal cavity. Additionally, the mucosa of the midline upper jaw was observed to be red and swollen, without any signs of ulceration (Fig. 2J). Plasma EBV-DNA load was 440 copies/ml. Nasopharynx enhanced CT revealed soft tissue thickening of nasal septum and lateral nasal wall, partial discontinuity of nasal septum bone (Fig. 2K), and irregular bone destruction in hard palate and upper alveolar (Fig. 2L). The final diagnosis was ENKTL with low-risk stage II disease based on above findings.

### Response and survival

All five patients completed the planned chemotherapy and radiotherapy according to the scheduled regimen. Following the completion of two cycles of chemotherapy and radiotherapy, four patients achieved a CR, and one patient demonstrated a partial response (PR). Case 1 was adjusted to G3 group due to EBV activation after received 4 cycles of chemotherapy. Subsequently, after the completion of six cycles of chemotherapy, allogeneic hematopoietic stem cell transplantation (Allo-HSCT) was carried out. By the end of the therapy, all patients achieved a complete response (CR). The patients were subsequently followed up until June 2022, with a median follow-up duration of 22 months (ranging from 5 to 57 months). At the conclusion of the follow-up period, four patients remained in CR, with event-free survival durations of 47, 31, 20, and 16 months, respectively. Out of the total number of patients, only one patient was not followed up, and their status remains unknown. The reason for the loss of follow-up in this patient was attributed to economic factors, as they reside locally but have been unable to undergo regular physical examinations. The median OS time was 33 months, ranging from 18 to 57 months, while the median EFS time was 20 months, ranging from 5 to 47 months.

### Toxicity

The most common adverse effects observed during chemotherapy were hematologic toxicities; however, no severe infections related to chemotherapy were reported. All five patients (100%) experienced grade 4 leukopenia, and three patients (60.0%) experienced grade 3 thrombocytopenia (Table 4). Severe non-hematologic toxicities were infrequent, with only one case (case 3) experiencing grade 4 oral mucositis. This was attributed to the presence of homozygous mutations in MTHFR (C677T) and SLO1B1 (rs4149056) genes (Fig. 1B). During radiotherapy (RT), toxicities were minimal, with only one patient experiencing grade 2 radiation-related dermatitis. There was no treatment-related death.

**Table 4:** Toxicity with regimen

Toxicity	Grade 1	Grade 2	Grade 3	Grade 4
Hematologic				
Neutropenia	0	0	0	5 (100%)
Thrombocytopenia	1 (20%)	1 (20%)	3 (60%)	0
Non-Hematologic				
Mucositis related to radiation	1 (20%)	0	0	0
Mucositis related to chemotherapy	1 (20%)	0	0	1 (20%)
Dermatitis related to radiation	0	1 (20%)	0	0
Allergy	0	0	0	0
Abnormal hepatic function	1 (20%)	1 (20%)	1 (20%)	0
Infection	0	0	2 (40%)	0

## Discussion

ENKTL is the second most common subtype of non-Hodgkin lymphoma (NHL) and accounts for 57.8% of mature T-cell and NK-cell lymphomas in adult [26]. Most clinical studies with large sample sizes are predominantly conducted in Asia, primarily due to the high incidence rate of the condition in the region. However, ENKTL in children is rare compared to adults. It is difficult to estimate a population-based incidence of ENKTL in children owing to the lack of national registry data. Several retrospective studies of children and adolescents from pediatric cancer centers show that NK/T-cell lymphomas constitute between 0.2% and 1.6% of newly diagnosed cases of NHL in children and adolescents [3].

Combination modalities of radiotherapy and chemotherapy have been advised in clinical practice including concurrent, sequential, and sandwich chemo-radiotherapy in ENKTL [27]. Historically, the use of CHOP or CHOP-like regimens resulted in 5-year overall survival rates of less than 50%. Subsequently, p-glycoprotein encoded by the multidrug resistance *(MDR) 1/ABCB1* gene is found to be frequently expressed in ENKL cells. The chemotherapy strategy has been changed from anthracycline-containing regimens to non-MDR-dependent therapies [28]. Ando M. et al. found that NK cells lack the asparagine synthase activity found in most normal cells, and asparaginase has been shown to induce apoptosis in NK/T-cell lymphoma cell lines in vitro. SMILE chemotherapy comprises L-asparaginase, two non-MDR-related agents (methotrexate and ifosfamide), etoposide, and dexamethasone, and is regarded as the standard regimen for ENKTL patients. However, severe adverse events remain an obstacle in clinical practice. Earlier investigations have indicated that approximately 70% of patients with ENKTL experience severe leukopenia (grades 3–4), leading to poor tolerance to treatment [29, 30]. Therefore, low-toxicity chemotherapy with radiotherapy, such as RT and a two-thirds dose of DeVIC (dexamethasone, etoposide, ifosfamide, and carboplatin) chemotherapy (RT-2/3DeVIC), CCRT-VIDL (etoposide, ifosfamide, dexamethasone, and L-asparaginase), and RT-P-GEMOX, is applied to the treatment of adult ENKTL [31, 32]. To date, the optimal chemotherapy regimen and the most effective combination of radiotherapy and chemotherapy have not been definitively established, particularly in the case of children with ENKTL. At present, few clinical studies with large sample were reported on ENKTL in children [33]. There is a lack of prospective data specifically focusing on the treatment of ENKTL in the pediatric population. Therefore, the management of ENKTL in children primarily relies on extrapolating clinical experiences and insights from adult cases.

Because children have no underlying illnesses and exhibit a high tolerance for chemotherapy, this research opted for the modified SMILE regimen as the fundamental structure of a sandwich protocol. The modified SMILE (mSMILE) regimen substitutes L-asparaginase with pegaspargase and reduces the cycle duration to 21 days. The efficacy and safety of the protocol were assessed in newly diagnosed children with EKNTL. The findings demonstrated that all patients experienced favorable remission, and the overall survival time reached to 33 months.

The common disadvantage of regimens containing SMILE is that the toxicity of treatment is high and that patients are not easily tolerated. Hematologic toxicity and infection were frequent and severe in adult ENKTL patients. In this study, all patients experienced grade 4 leukopenia and 3 of 5 patients had grade 3 thrombocytopenia. However, the hematologic toxicity resolved rapidly due to the absence of underlying illnesses, and no deaths occurred as a result of the treatment. Only 1 patient had grade 4 oral mucositis. To identify the underlying causes, the gene polymorphisms and hot spot mutations of related methotrexate metabolic enzymes were detected. The patient was discovered to possess both the MTHFR (C677T) and SLO1B1 (rs4149056) genetic variants. As a result, in the subsequent chemotherapy sessions, the methotrexate dosage was decreased by 20%, and regular monitoring of serum drug levels ensured they remained within the appropriate range. Consequently, the patient did not experience severe oral mucositis again. The results confirmed that mSMILE regimens with sandwiched radiotherapy yielded promising outcomes in the treatment of ENKTL in children/adolescents in our center. Due to small cases in this study, we have carried out a phase II multicenter clinical trial (ChiCTR220005954) for children with ENKTL in China to further verify the efficacy and safety.

Hematopoietic stem cell transplantation (HSCT) holds great promise as a novel therapeutic option for cancer. However, the role of HSCT in the treatment of ENKTL is controversial. The knowledge and expertise regarding HSCT in ENKTL are still limited [34]. Several small, retrospective studies suggest that consolidative HSCT is benefit in advanced-stage or relapsed/refractory ENKTL who achieved complete remission after chemotherapy [17]. Moreover, the most appropriate type of HSCT (autologous or allogeneic) for ENKTL patients, as well as the ideal timing (whether upfront or as salvage therapy), remains debatable [35, 36]. The retrospective study revealed that autologous HSCT has limited advantages in treating advanced or relapsed ENKTL. Autologous HSCT is more preferred for disseminated ENKTL who achieve first CR. The allogeneic HSCT should be used in advanced patients who fail to achieve a second CR after relapse [37–39]. There is a general agreement that consolidative HSCT is not required for newly diagnosed localized ENKTL patients who have achieved a complete response following chemo-radiotherapy. The upfront use of HSCT in ENKTL is linked to an extremely unfavorable prognosis [40, 41]. In our study, one patient received allogeneic HSCT (Allo-HSCT) who had EBV activation after 4 cycles of chemotherapy. After the completion of six cycles of chemotherapy, the patient underwent HSCT. By the end of the treatment, the patient achieved CR.

Genetic abnormalities, EBV infection, and tumor microenvironment (TME) have been shown to play important roles in the molecular pathogenesis of ENKTL. However, it is often difficult to obtain sufficient specimens due to destructive lesions for genetic analysis in clinical practice. Therefore, no relevant analysis was conducted in this study. Next-generation sequencing (NGS) technology data from clinical studies have revealed the Janus kinase/signal transducer, and activator of transcription (JAK/STAT) pathway is crucial for NK cell development and maturation. The JAK/STAT pathway, known for its role in promoting cell proliferation, is primarily associated with activating mutations in the NK lineage, and constitutive activation of this pathway has been reported in approximately 12.4–35% of ENKTL cases [13, 42].

According to various studies, STAT3 mutations are the most commonly observed genetic alterations, followed by activating mutations in JAK3. In the treatment of ENKTL, there are multiple inhibitors available that target the JAK/STAT pathway [15]. The selective JAK3 inhibitor has recently been demonstrated to have a potent anti-tumor activity in pre-clinical study. In pre-clinical research, it has been shown that the JAK1/2 inhibitor ruxolitinib can effectively disrupt JAK/STAT signaling in ENKTL cases with STAT3 mutations or overexpression [16, 43, 44]. Immune evasion is an additional mechanism that contributes to the development of NK/T-cell lymphoma. In ENKTL, Epstein-Barr virus (EBV) infection induces the excessive expression of programmed death protein ligand 1 (PD-L1), which can aid tumor cells in evading immune surveillance by suppressing T cell activation, proliferation, cytokine secretion, and survival [44]. The positive rate of PD-L1 was reported to be 39–100% in ENKTL tissues [16]. Anti-PD-1 antibodies (pembrolizumab, nivolumab, sintilimab, tislelizumab, and avelumab) have been studied in small cohorts of relapsed or refractory ENKTL, and have shown promising results [15, 45–47]. The 2018 National Comprehensive Cancer Network (NCCN) guidelines recommend pembrolizumab and nivolumab for relapsed/refractory ENKTL [17, 48, 49]. Multiple studies have demonstrated a correlation between serum PD-L1 levels and the prognosis of ENKTL. Consequently, PD-1 blockade is being investigated as a potential treatment option for newly diagnosed advanced-stage disease. An ongoing clinical trial (NCT03719105) is evaluating the effectiveness of the modified SMILE chemotherapy regimen alone, as well as in combination with pembrolizumab, in children, adolescents, and young adults with advanced-stage NK lymphoma and leukemia. Allo-HSCT will be performed according to disease response. Until now, no relevant data has been reported. Similar to this clinical study, PD-1 inhibitor combined with the mSMILE regimen and Allo-HSCT is also attempted to be used for the treatment of high-risk children ENKTL in our clinical trial ChiCTR220005954. Initial results (unpublished data) have shown encouraging results. However, the long-term follow-up is necessary to assess if responses are durable. Moving forward, determining the optimal combinations of these treatments and identifying the most effective combinations for pediatric patients will be crucial clinical considerations. In summary, pediatric ENKTL is uncommon compared to its occurrence in adults, and precise clinical epidemiological data is currently lacking. The standard first-line regimens are yet to be determined, especially in children with ENKTL. Although the SMILE regimen is associated with significant hematologic toxicity and poor tolerance in adult ENKTL patients, it is better tolerated and yields favorable treatment responses in children with ENKTL due to the absence of underlying diseases. Immune blockade of the PD1/PD-L1 axis might be effective treatment for advanced-state ENKTL. Multicenter clinical trials should be established to guide future treatment in the era of precision medicine.

## Data Availability

The original contributions presented in the study are included in the article/supplementary material. Further inquiries can be directed to the corresponding author.
